# Qualitative and Quantitative Analysis for the Chemical Constituents of* Tetrastigma hemsleyanum* Diels et Gilg Using Ultra-High Performance Liquid Chromatography/Hybrid Quadrupole-Orbitrap Mass Spectrometry and Preliminary Screening for Anti-Influenza Virus Components

**DOI:** 10.1155/2019/9414926

**Published:** 2019-02-18

**Authors:** FuJuan Ding, JiangTing Liu, RuiKun Du, QinHui Yu, LiLi Gong, HaiQiang Jiang, Rong Rong

**Affiliations:** ^1^School of Pharmacy, Shandong University of Traditional Chinese Medicine, Jinan, Shandong, China; ^2^Collaborative Innovation Center for Antiviral Traditional Chinese Medicine, Shandong Province, China; ^3^Key Lab for Natural Product, Shandong Province, China

## Abstract

*Tetrastigma hemsleyanum* Diels et Gilg (*T. hemsleyanums*) is a kind of traditional folk medicinal plant which has been used widely in China for its antivirus, antitumor, and other clinical effects. In this study, ultra-high performance liquid chromatography coupled with hybrid quadrupole-orbitrap mass spectrometry (UPLC-Q-Exactive/MS) was utilized to analyze the chemical constituents of* T. hemsleyanums*. Fifty-one constituents were clarified, including flavonoids, anthraquinones, esters, fatty acids, phenols, and catechins. In the subsequent quantitative analysis, the contents of ten compounds of rutin, kaempferol, astragalin, quercitrin, quercetin, vitexin-rhamnoside, isorhamnetin, vitexin, emodin-8-O-*β*-D-glucoside, and isoquercetin in 18 batches of* T. hemsleyanums* collected from different places of cultivation were determined. Meanwhile, anti-influenza virus bioactivity in vitro of the above samples was detected with Gaussia Luciferase viral titer assay. It was found that the antiviral bioactivity varied from batches to batches in accordance with content difference of the chemical constituents in* T. hemsleyanums*. Correlation analysis was performed with SPSS software for the association between LC-MS chemometrics and bioactivity of influenza virus inhibition, and 8 constituents of flavonoids showed positive correlation coefficient, which may provide a valuable clue for searching potential antiviral components in* T. hemsleyanums*.

## 1. Introduction

*Tetrastigma hemsleyanum* Diels et Gilg (abbreviated. as* T. hemsleyanums*) is a kind of medicinal and edible plant wildly growing in south China, especially in Zhejiang, Guangxi, and Yunnan provinces [1]. It was firstly recorded in* Zhong Hua Ben Cao* [2] for its effects of clearing away heat, detoxifying, removing phlegm, promoting blood circulation, and relieving pain. Nowadays, it is used in Traditional Chinese Medicine (TCM) clinics for the treatment of children with high fever (convulsions), viral meningitis, pneumonia, and hepatitis. Many literatures confirmed that* T. hemsleyanums* belongs to the folk medicine; its root can be used to treat children's wind-heat with water decoction which is recorded in local standardization, such as “*Guangxi min jian chang yong shou yi cao yao*” [3], “*Kunming min jian chang yong cao yao*” [4], and “*Zhejiang min jian chang yong cao yao*” [5]. The modern pharmacological studies had shown that it had effects of anti-inflammatory, analgesic, antipyretic, antivirus, antitumor [6–8], immunomodulatory [9], liver protection, etc.

In recent years researchers have paid more and more attention to flavonoids and anthraquinones which wildly exist in Chinese herbal medicine because of their outstanding bioactivities. Modern phytochemical studies have shown that flavonoids are the major active constituents in* T. hemsleyanums* [10]. Out of them, vitexin, isoquercetin, and astragalin showed the anticancer effect [11–13], quercitrin could improve the impaired mesenteric vascular activity in the colitis models [14], quercetin could yield an obvious mitigation of arthritic manifestations [15], kaempferol had effects of preventing and reversing ventricular fibrosis and cardiac dysfunction* in vivo* and* in vitro* experiments [16], and rutin played a therapeutic role in diabetic atherosclerosis through inhibiting premature senescence of vascular smooth muscle cells [17]. Because of their prominent pharmacological activities, determination for the contents of the above compounds in* T. hemsleyanums* and also the LC-MS characterizations of the plant are required for further quality control study.

It was reported recently that UPLC-Triple-TOF/MS had been applied for the qualitative analysis of the chemical compositions in* T. hemsleyanums*, and most of them were identified as flavonoids, esters, and benzene sulfonic acids [18]. UPLC-Q-Exactive/MS is a more powerful and sensitive analytical instrument for detecting constituents even in low abundance in complex plant extracts [19]. In this present study we use UPLC-Q-Exactive/MS technique firstly to analyze the components of* T. hemsleyanums* collected from different cultivation places in China. The data generated from UPLC-Q-Exactive/MS have distinguished the phytochemical differences among the 18 batches both in types of ingredients and in contents. Various factors, such as cultivation places, harvest season, and postharvest treatment, are responsible for the variation in contents of phytochemicals and hence are due to the variation of many bioactivities.

We performed the antiviral determination assay using recombinant influenza virus PR8-NS1-Gluc to evaluate the anti-influenza virus bioactivity of the extracts from* T. hemsleyanums*. It is a high throughput screening protocol to identify entry inhibitors for influenza virus using a human immunodeficiency virus-based pseudotyping platform which can be performed in a BSL-2 facility. Conventional methods of active compound discovery from natural products involve bioactivity-guided isolation, which is laborious, costly, or impossible to source. In addition, the effects of underlying constituents with potent bioactivity present at very low or sometimes undetectable levels may be ignored. In this study, we process the data with SPSS correlation analysis to evaluate the association between chemometrics and bioactivities of the 18 batches of* T. hemsleyanums* from different districts. Content of 8 constituents of flavonoids showed positive correlation coefficient with anti-H1N1 influenza virus activity, which may facilitate the identification of potential antiviral components in* T. hemsleyanums*.

## 2. Materials and Methods

### 2.1. Chemicals and Plant Materials

HPLC-grade acetonitrile and formic acid were purchased from Thermo Fisher Scientific Company (USA). Ultra-pure water was purchased from Watsons Company (China). All other reagents were of analytical grade.

The reference standards such as kaempferol, isoquercetin, astragalin, rutin, quercetin, baicalein, and palmitic acid were purchased from Herbpurify Co., Ltd. (Chengdu, China); emodin-8-O-*β*-D-glucoside, quercitrin, vitexin, and vitexin-rhamnoside were purchased from Yuanye Bio-Technology Co., Ltd. (Shanghai, China); isorhamnetin, catechins and epicatechin were purchased from Institutes for Food and Drug control (Beijing, China).

The plant materials of* T. hemsleyanums* were collected from different provinces in China, as Zhejiang (ZJ1, ZJ2, and ZJ3), Guangxi (GX1, GX2, and GX3), Yunnan (YN1, YN2, and YN3), Fujian (FJ1, FJ2, and FJ3), Guizhou (GZ1, GZ2, and GZ3), and Hubei (HB1, HB2, and HB3). The botanical authentication was performed by Professor Lingchuan Xu from Department of Pharmacognosy, Shandong University of Traditional Chinese Medicine. A voucher specimen (no. TH20171201~TH20181118) was deposited in Key Lab for Natural Product, Shandong Province. The dried rhizome parts of* T. hemsleyanums* were used for further analysis.

### 2.2. Liquid Chromatography and Mass Spectrometry Analysis

In this study we employed a UPLC system tandem Q-Exactive/MS spectrometer (Thermo Fisher, CA, USA) equipped with a heated electrospray ionization (HESI) probe. Halo C18 (2.7 *μ*m, 100 × 2.1 mm, Advanced materials technology, USA) column was used at the flow rate of 0.3 mL/min and column temperature of 30°C. The binary solvent system consisted of 0.05% aqueous formic acid (v/v) (A) and 0.05% formic acid in acetonitrile (B). Samples were eluted with the following linear gradients: 15% B at 0–15 min; 15–40% B at 15–22 min; 40–90% B at 22–50 min; 90–15% B at 50–60 min. Injection volume was 3 *μ*L. Detection was performed using a Q-Exactive™ hybrid quadrupole-Orbitrap mass spectrometer in both positive and negative ionization modes. The optimal analysis conditions were set as follows: ion source, heated electrospray ionization probe; source temperature: 350°C; capillary temperature: 320°C; sheath gas: 45 arb; auxiliary gas: 10 arb; mass collecting range: m/z 100-1500. The full scan and fragment spectra were collected at the resolutions of 70000 and 17500, respectively. The collision energy was 30 eV, 50 eV, and 70 eV at negative mode and 10 eV, 30 eV, and 50 eV at positive mode.

### 2.3. Sample Preparation

The dried rhizome parts of* T. hemsleyanums* were ground to powder. For each sample, 15 g of rhizome powder was extracted with 75% ethanol reflux for 3 times, 1 h each time. The ethanol extracts were concentrated under reduced pressure evaporated to dryness and then dissolved with 50% acetonitrile of 25.0 mL as the stock sample solution. 1.0 mL of the above stock sample solution, adding baicalein with the final concentration of 4.781 *μ*g/mL as the internal standard (IS), was filtered through a 0.22 *μ*m syringe filter to obtain sample solution for qualitative and quantitative analysis. 20.0 mL of the stock sample solution was freeze-dried in vacuum to obtain a sort of powder extract for antiviral bioactivity testing.

### 2.4. Preparation of Standard Solutions

Rutin, kaempferol, astragalin, quercitrin, quercetin, vitexin-rhamnoside, isorhamnetin, vitexin, emodin-8-O-*β*-D-glucoside, and isoquercetin were dissolved in HPLC-grade acetonitrile to achieve standard stock solution with the concentration of 1.0 mg/mL and serially diluted to mixed working solution with 50% acetonitrile; baicalein was used as IS with concentration of 4.781 *μ*g/mL in working solution. All the stock and working solutions were stored at 4°C.

### 2.5. Identification of the Constituents

LC-MS data were acquired in both negative and positive ion modes and processed for target compounds identification using a combination of Xcalibur and Mass Frontier software packages (Thermo Scientific).

### 2.6. Validation of Quantitative Method

10 constituents of flavonoids were chosen for the content measurement. Internal standard method was used for quantitation. Peak area ratios of the analyte against IS were used for calculations and a weighted (1/concentration) regression analysis was used for standard curves preparation. Limit of detection (LOD) and limit of quantification (LOQ) for each constituent were detected. The intraday and interday precision of the determination for ten constituents were validated. Stability of sample solutions within 24 hours at 4°C was tested. Six replicates of samples were prepared to check the repeatability. The recoveries were analyzed by adding the analytical reference standards in the powder of* T. hemsleyanums* prior the extraction procedure and assessed at three levels of the amount which measured in analyte to standards added (1:0.8, 1:1, 1:1.2), using six replicates at each level.

### 2.7. Quantitative Analysis for Ten Compounds of Flavonoids

Contents measurement of ten compounds of flavonoids in 18 batches of* T. hemsleyanums* was performed under the condition of Section 2.2.

### 2.8. Anti-H1N1 Influenza Viral Determination

The antiviral determination assay was performed using recombinant influenza virus PR8-NS1-Gluc as previously described [20]. Briefly, MDCK cells grown in 24 well plates were inoculated with recombinant influenza virus PR8-NS1-Gluc at an moi of 0.01 PFU/cell. After incubation for 1 h at 37°C, virus inoclula were removed and cells were washed. Opti-MEM containing 2 *μ*g/mL TPCK-trypsin was then added for virus propagation. At 36 hours after infection (hpi), 50 *μ*l of culture medium was removed for luciferase assay using BioLux Gaussia Luciferase Assay kit (NEB, USA) according to the manufacturer's instructions. For antiviral activity determination, extracts of* T. hemsleyanums* were added at indicated concentrations as shown in Figure 5 during virus propagation.

### 2.9. Statistics

In order to study the association between the chemical spectra and the anti-influenza viral bioactivity, correlation analysis was performed using the SPSS 17.0 software. The two variables in the correlation analysis were the ion intensity and the inhibitory effect against H1N1 influenza virus. All statistical analyses were two sided. p<0.05 was considered to be statistically significant.

## 3. Results and Discussion

### 3.1. Qualitative Analysis

**Figure 1:**
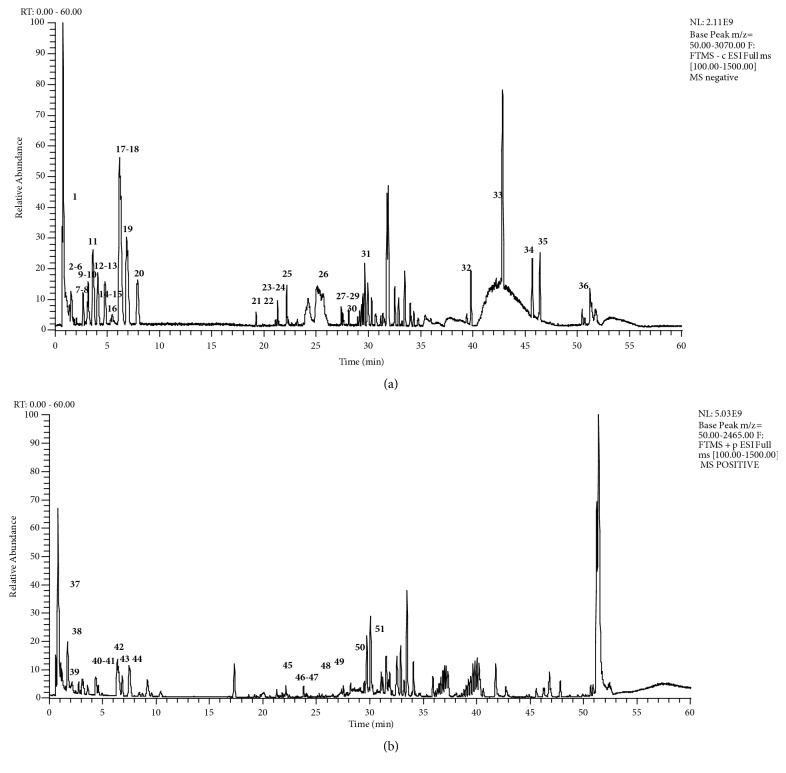
Ultra-high performance liquid chromatography coupled with hybrid quadrupole-orbitrap mass spectrometry (UPLC-Q-Exactive/MS) was utilized to analyze the chemical constituents of* T. hemsleyanums*. The UPLC-Q-Exactive/MS base peak chromatogram of* T. hemsleyanums* in negative (a) and positive (b).

Sample solution of* T. hemsleyanums* collected from Guangxi province was chosen for qualitative analysis. UPLC-Q-Exactive/MS base peak chromatograms of* T. hemsleyanums* were shown in Figure 1, and the identification results in negative ion mode and positive ion mode of mass spectrometry were shown in Tables 1 and 2. As shown in the tables, fifty-one compounds were clarified including flavonoids, anthraquinones, esters, fatty acids, phenols, and catechins according to accurate molecular weight calculation, ion fragmentation information, and some of them with confirmation of reference standards.

Flavonoids are mainly as follows: compounds 1, 8, 10, 12, 14, 18, 19, 20, 21, 24, and 25 were assigned to be procyanidin dimmer, isorhamnetin-3-pyranosearabinose-7-glucosyl-rhamnoside, kaempferol-3-O-furananose-7-O-rhamnosyl-glucoside, rutin, isoquercetin, kaempferol-3-O-rutone, astragalin, quercetin, vitexin, kaempferol, and isorhamnetin [18], and compound 17 was assigned to be quercitrin [21]. Compounds 11 and 21 were assigned to be vitexin-rhamnoside [21] and vitexin [22]. Compound 21 was also detected in positive ion mode. Compound 49 was the isomer of compound 21. Compounds 42 and 44 were assigned to be orientin [23] and isoorientin [24]. Peak 15, kaempferol-3-O-furanosine-7-O-rhamnose isomer, was detected from* T. hemsleyanums *for the first time.

**Figure 2:**
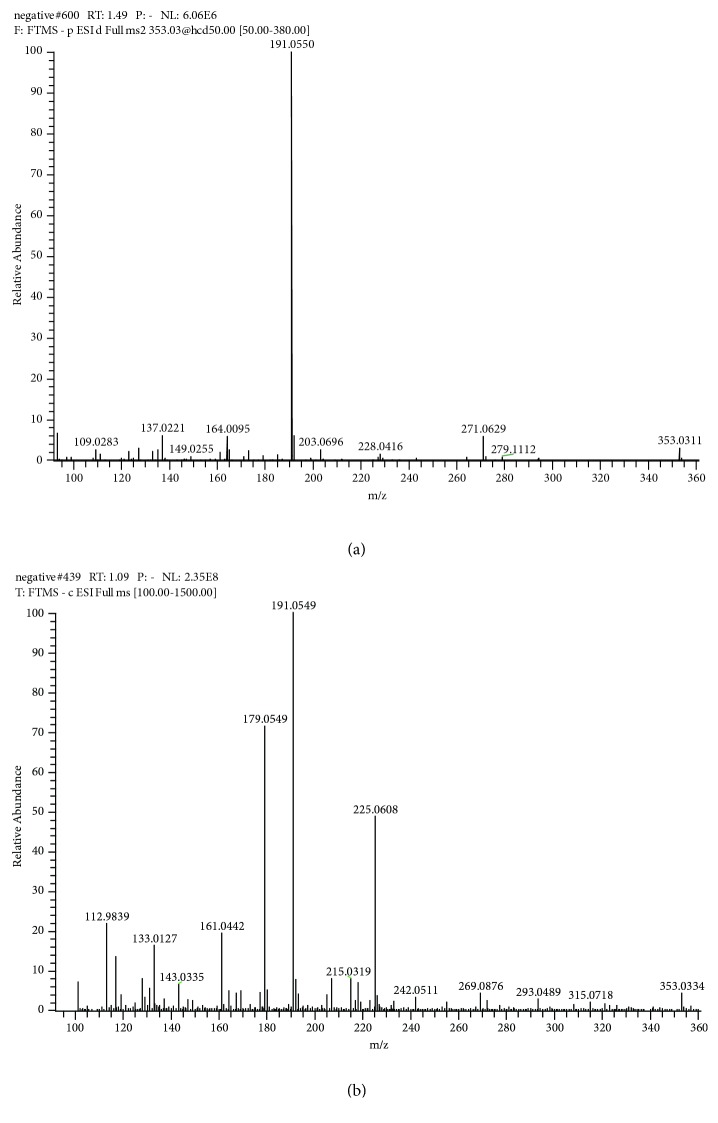
Secondary mass spectrum of chlorogenic acid (a) and neochlorogenic acid (b). As shown in (a), the cleavage fragment strength of the m/z was 191> 179>135. In (b), the peak m / z = 191 has the strongest intensity, and the peaks m / z179 and m / z135 have the same intensity and are very weak.

Phenolic acids are mainly as follows: compounds 2 and 3 were assigned to be neochlorogenic acid and chlorogenic acid [22]. As we saw in Figure 2, the cleavage fragment strength of the neochlorogenic acid was m/z=191>179>135 (Figure 2(a)). And for chlorogenic acid, the strength of peak m/z=191 was the strongest; the strength of peaks m/z 179 and m/z 135 was equivalent and very weak (Figure 2(b)).

**Figure 3:**
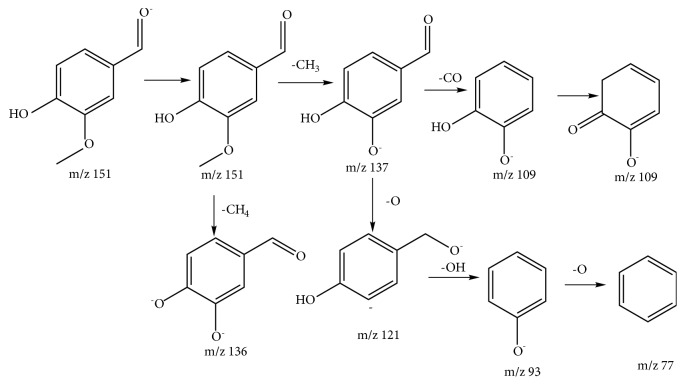
A proposed fragmentation pathway of 4-hydroxy-3-methoxybenzaldehyde by mass spectrometry.

Compound 9 was assigned to be 4-hydroxy-3-methoxybenzaldehyde, of which fragmentation patterns was shown in Figure 3. Compounds 16 and 26 were assigned to be salicylic acid and rock acid [18].

**Figure 4:**
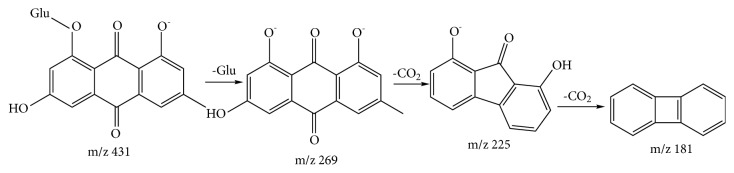
A proposed fragmentation pathway of emodin-8-O-*β*-D-glucopyranoside by mass spectrometry.

Anthraquinones are mainly as follows: compound 22 was assigned to be emodin-8-O-*β*-D-glucopyranoside [25]. The [M-H]^−^ ion of compound 22 was at m/z 431.1557. Glucose group was lost to produce ion of m/z 269.1029. The fragmentation patterns were summarized in Figure 4.

Catechins are mainly as follows: compounds 27 and 29 were assigned to be gallocatechin and epigallocatechin [23]. Compound 29 produced characteristic ions of m/z 125.0957 and m/z 137.0593 which was identified as epigallocatechin. Compound 27 and compound 29 were also detected in positive ion mode. Compounds 4, 5, 6, and 7 were assigned to be protocatechuic acid glucoside, gallic acid, catechins, and epicatechin [26, 27]. Catechins and epicatechin were confirmed by reference standard.

Esters are mainly as follows: compounds 28, 30, 32, 34, 35, and 51 were assigned to be gingergly eolipid A, gingergly eolipid B, lysophosphatidic acid, linoleic acid phosphatidic acid, 4-(2-dodecyl)-benzene-sulfonate, and methyl linolate [18].

Fatty acids are mainly as follows: compounds 39 and 50 were assigned to be palmitic acid and linoleic acid. Additionally, the loss of H_2_O group was considered as the representative fragmentation pathway in acids [28]. Palmitic acid was confirmed by reference standard.

The other compounds are mainly as follows: compounds 31, 33, and 36 were assigned to be 1-linoleoylglycero-2-phosphor-ethanolamine, soya-cerebroside I, and 4-(1-methyl-dodecyl)-benzene-sulfonic acid [18].

### 3.2. Quantification Method Validation

As shown in Table 3, standard curves of the ten compounds exhibited good linearity in the range of 1.6 to 1330 *μ*g/mL, with coefficients of correlation ranging from 0.9961 for kaempferol to 0.9998 for quercetin. The parameters of LOD were from 0.1045 to 2.0781 pg, and LOQ were from 0.9290 to 15.5142 pg, which were sensitive enough to the detection of analytes. The intraday and interday precisions of present method were shown in Table 4, and the respective RSD values were from 0.03 to 2.79% and from 0.72 to 2.81%. The concentration stability of the constituents in samples kept at 4°C for 24 hours (n = 6) ranged from 0.28 to 3.42%. In addition, the sample solutions for* T. hemsleyanums* were prepared in parallel (n=6) to evaluate the repeatability and achieved the RSD of 0.26-2.69%. Based on the above methodology verification, the assay is reproducible and suitable for accurate and precise quantification of these ten chemical constituents in* T. hemsleyanums *(shown in Table 4). As shown in Table 5, the recoveries of ten constituents was from 94.86 to 99.9 % with the RSD value <3 %.

### 3.3. Quantitative Analysis

The validated UPLC-Q-Exactive/MS method was used for the content measurement of 10 constituents of rutin, kaempferol, astragalin, quercitrin, quercetin, vitexin-rhamnoside, isorhamnetin, vitexin, emodin-8-O-*β*-D-glucoside, and isoquercetin in* T. hemsleyanums*. The content of each constituent was calculated in terms of its respective calibration curve and the result was listed in Table 6. The content of vitexin was relative abundant, of which could reach the amount of 0.7079±0.0286 mg/g in that from YN1, whereas vitexin-rhamnoside may be the lowest in content since it could even not be detected in some batches. The result suggested that contents of these constituents fluctuated in different batches even for those that from the same production place. The total amounts of these ten compounds in* T. hemsleyanums* of YN1, GZ1, and GX3 were the top three highest, whereas for samples from Zhejiang province, amounts of flavonoids are shown to be the lowest. Many factors including growing environment of climate temperature, rainfall, sunlight, soil nutrient, etc. and harvest time and postharvest treatment may affect the content of bioactive compounds in* T. hemsleyanums* and thus may affect its efficacy.

### 3.4. Anti-H1N1 Influenza Virus Activity

**Figure 5:**
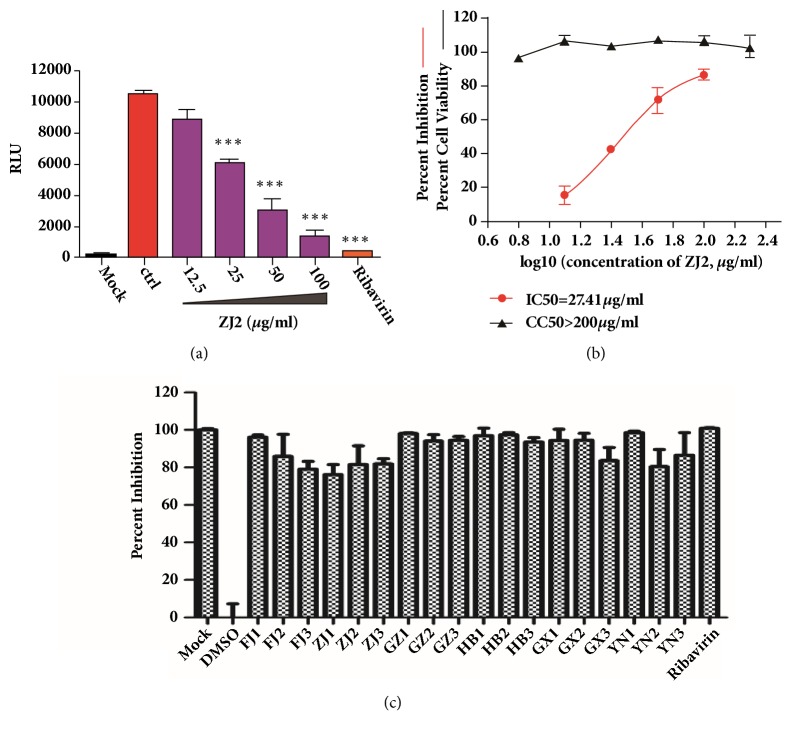
Antiviral determination. MDCK cells were infected with recombinant influenza virus PR8-NS1-Gluc at an moi of 0.01 PFU/cell, and the infected cells were treated with ZJ2 and other extract samples of* T. hemsleyanums* at indicated concentrations during virus propagation. (a) ZJ2 inhibits influenza virus replication in a dose dependent manner. (b) Dose-response curve of ZJ2 against influenza virus replication and on cell viability. (c) 17 extract samples of* T. hemsleyanums* collected from different districts as well as ZJ2 show similar antiviral activities against influenza virus. Mean and SEM of three independent experiments were shown. *∗∗∗*p<0.001, Student's t-test. There are 18 batches of* T. hemsleyanums* in this figure. Among them, FJ1, FJ2, and FJ3 are three batches from Fujian, GZ1, GZ2, and GZ3 are from Guizhou, ZJ1, ZJ2, and ZJ3 are from Zhejiang, HB1, HB2, and HB3 are from Hubei, YN1, YN2, and YN3 are from Yunnan, and GX1, GX2, and GX3 are from Guangxi. Their information can be found in the Supplementary Materials (available here).

Since* T. hemsleyanums *from Zhejiang province is popular on market, we took sample of ZY2 as the representative sample for anti-influenza virus pretest. As shown in Figure 5(a), extract of ZJ2 inhibited influenza virus replication in a dose-response manner, and the IC50 is 27.4*μ*g/mL, while no cytotoxicity was observed at a concentration of as high as 200*μ*g/mL (Figure 5(b)). Ribavirin at 100*μ*M was used as positive control. Antiviral activities of the other 17 batches were shown in Figure 5(c) that all of them showed inhibitory effect against H1N1 influenza virus replication as good as ZJ2 at the concentration of 50*μ*g/mL. All three sample batches from Guizhou province (GZ1, GZ2, and GZ3, 94.0-98.0%) and Hubei province (HB1, HB2, and HB3, 93.5-97.3%), two batch from Guangxi province (GX1 and GX2, 94.3-94.5%), one batch from Fujian (FJ1, 96.2%), and one batch from Yunnan (YN1, 98.6%) exhibited higher anti-influenza virus activities than the others, which were probably due to the higher values of flavonoids in these samples.

### 3.5. Statistical Analysis

In order to determine the contribution of various constituents for the antiviral activity, the spectrum-effect relationships between LC-MS fingerprints and inhibitory effect for influenza virus were evaluated with correlation analysis statistical method. Since normal distribution was not satisfied, Spearman was used for all the analyses. When the correlation coefficient is greater than 0, it indicates that a component is positively correlated with antiviral activity, and the larger the value, the stronger the correlation. When the correlation coefficient is less than 0, it means that it is negatively related to antiviral activity. Results were shown in Table 7 which revealed that 10 peaks had significant correlationship with anti-influenza virus activity. 8 of them, which were identified as rutin, kaempferol, astragalin, quercitrin, quercetin, kaempferol-3-o-rutinoside, procyanidin dimmer, and epicatechin, were positively related to antiviral activity, of which the highest correlationship was with astragalin (r = 0.711*∗∗*), followed by epicatechin (0.641*∗∗*), quercitrin (0.614*∗∗*), and quercetin (0.617*∗∗*). 2 of the 10 peaks, identified as vitexin (-0.5370*∗∗*) and isorhamnetin-3-pyranose arabinose-7-glucosyl rhamnoside (-0.6630*∗∗*), indicated negative correlationship with antiviral activity. That is, for constituents with positive coefficient, which may have underlying anti-H1N1 influenza effect, the higher the content, the better the bioactivity. This result was in agreement with literatures, in which quercetin, quercitrin, rutin, kaempferol, and isoquercitrin were reported to have anti-H1N1 influenza virus activity [29–32]. Thereby it may provide valuable clues for further discovery of active anti-influenza virus compounds. LC-MS chemometrics are a kind of potent method for analyzing the complex system of TCM herbs, but lacking of bioactive characteristics. Therefore, chemical fingerprints combined with bioactivity evaluation to construct a fingerprint-efficacy relationship is a good way to explore the bioactive constituent of TCM herb and may lend material basis supporting to the quality control of* T. hemsleyanums*

## 4. Conclusion

In our study, an efficient UPLC-Q-Exactive/MS method was established for qualitative analysis of up to 51 constituents identified in* T. hemsleyanums* for the first time and also for quantitative analysis of 10 constituents in 18 batches collected from six cultivation places. Method validation showed the ideal sensitivity, stability, and reliability of the analysis method. It was also the first time for UPLC-Q-Exactive/MS and Gaussia Luciferase viral titer assay being combined to reveal the underlying anti-influenza virus bioactive compounds in* T. hemsleyanums*. 8 possible active constituents were discovered showing positive contribution for antivirus effect, and five of them are consistent with reports published in literatures. This analytical method led to the identification of compounds with anti-H1N1 influenza virus activity in* T. hemsleyanums* which may be difficult to be extracted and identified by traditional bioactivity-guided fractionation procedures and may provide an available reference mode for revealing the material basis in traditional Chinese herb of which thereby could also provide reference for quality assessment of TCM herbs.

## Figures and Tables

**Table 1:** Identification analysis of chemical constituents of *T. hemsleyanums* in negative ion mode of mass spectrometry.

No.	tR (min)	Name	Formula	Detected m/z	Expected m/z	M - X	Error (ppm)	Fragmentor information
1	0.9	Procyanidin dimmer	C_30_H_26_O_12_	577.1334	577.1341	M – H	-0.0007	125.0228, 151.0386
								161.0230, 203.0706
								289.0716, 407.0767
2	1.15	Neochlorogenic acid	C_16_H_18_O_9_	353.0331	353.0867	M – H	-0.0536	135.0434, 161.0591
								173.0598, 179.0337
								191.0553
3	1.49	Chlorogenic acid	C_16_H_18_O_9_	353.0337	353.0867	M – H	-0.0530	135.0436, 161.0231
								173.0443,191.0549
4	1.5	Protocatechuic acid glucoside	C_13_H_16_O_9_	315.0717	315.0711	M – H	0.0006	108.0200, 109.0279
								152.0100, 153.0179
5	1.52	Gallic acid	C_7_H_6_O_5_	169.0119	169.0132	M – H	-0.0013	125.0228, 145.3992
								151.0022
6	1.67	Catechins	C_15_H_14_O_6_	289.2617	289.2613	M – H	0.0004	121.0280, 125.0230
								137.0231, 161.0597
								187.0391, 245.0817
7	2.04	Epicatechin	C_15_H_14_O_6_	289.2618	289.2613	M – H	0.0005	125.0228, 161.0595
								179.0337, 187.0391
								245.0813, 271.0609
8	2.74	Isorhamnetin-3-pyranosearabinose-7-glucosyl-rhamnoside	C_33_H_40_O_20_	755.6536	755.6526	M – H	0.0010	299.0200, 313.0343
								314.0414, 609.1453
9	2.93	4-hydroxy-3-methoxybenzaldehyde	C_8_H_8_O_3_	151.0389	151.0390	M – H	-0.0001	77.0397, 93.0328
								109.0279, 121.0275
								136.0150, 137.0217
								151.0389
10	3.68	Kaempferol-3-o-furananose-7-O-rhamnosyl-glucoside	C_32_H_38_O_19_	725.6232	725.6266	M – H	-0.0034	255.0300, 283.0253
								284.0330, 285.0393
								575.1413
11	4	Vitexin-rhamnoside	C_27_H_30_O_14_	577.5144	577.5124	M – H	0.0020	283.0609, 311.0560
								341.0664, 353.0664
								413.0873, 457.1156
12	4.18	Rutin	C_27_H_30_O_16_	609.5162	609.5112	M – H	0.0050	211.0394, 255.0299
								256.0332, 283.0238
								301.0348
13	4.23	Kaempferol-3-O-furana-nose-7-O-rhamnose isomer	C_32_H_38_O_19_	725.6237	725.6266	M – H	-0.0029	255.0295, 284.0325
								431.1031
14	4.86	Isoquercetin	C_21_H_20_O_12_	463.3790	463.3697	M – H	0.0093	151.0025, 243.0296
								255.0298, 271.0250
								300.0278
15	5.5	Oxidized resveratrol	C_14_H_12_O_4_	243.0656	243.0652	M – H	0.0004	157.0644, 160.0471
								175.0752, 197.0597
								201.0548, 225.0549
16	6.27	Salicylic acid	C_7_H_6_O_3_	137.0229	137.0233	M – H	-0.0004	93.0329, 108.0198
								119.7794
17	6.53	Quercitrin	C_21_H_20_O_11_	447.3834	447.3703	M – H	0.0131	151.0029, 255.0296
								301.0356, 426.9522
18	6.94	Kaempferol-3-O-rutino-side	C_27_H_30_O_15_	593.5119	593.5118	M – H	0.0001	255.0299, 267.0291
								285.0405
19	7.9	Astragalin	C_21_H_20_O_11_	447.3738	447.3703	M – H	0.0035	227.0346, 255.0299
								284.0329, 285.0398
20	19.27	Quercetin	C_15_H_10_O_7_	301.0352	301.0343	M – H	0.0009	121.0279, 149.0229
								151.0022, 161.0227
								178.9973, 245.0449
21	19.77	Vitexin	C_21_H_20_O_10_	431.0972	431.0973	M – H	-0.0001	269.0262, 283.0627
								311.0527, 341.0764
22	20.12	Emodin-8-O-*β*-D-glucoside	C_21_H_20_O_10_	431.1557	431.0973	M – H	0.0584	181.0859, 225.1121
								269.1029, 413.1434
23	20.98	Apigenin	C_15_H_10_O_5_	269.0454	269.0445	M – H	0.0009	159.0439, 181.0648
								213.0542, 225.0545
								227.0343, 241.0497
24	21.4	Kaempferol	C_15_H_10_O_6_	285.2303	285.2295	M – H	0.0008	159.0440, 183.0448
								211.0394, 227.0340
								239.0346
25	21.64	Isorhamnetin	C_16_H_12_O_7_	315.2507	315.2555	M – H	-0.0048	151.0023, 163.0020
								271.0254, 283.0234
								300.0273
26	22.19	Rock acid	C_18_H_14_O_8_	357.0607	357.0605	M – H	0.0002	112.6701, l21.0279
								163.0184
27	27.41	Gallocatechin	C_15_H_14_O_7_	305.2661	305.2607	M – H	0.0054	125.0962, 167.5970
								287.1647
28	27.62	Gingergly eolipid A	C_33_H_56_O_14_	675.3498	675.3586	M – H	-0.0088	161.0440, 277.2170
								397.1349, 415.14514
29	27.9	Epigallocatechin	C_15_H_14_O_7_	305.0750	305.0656	M – H	0.0094	125.0957, 137.0593
								179.1062, 287.1654
30	29.26	Gingergly eolipid B	C_33_H_58_O_14_	677.3754	677.3743	M – H	0.0011	125.0230, 161.0444
								179.0553, 279.2327
								397.1341, 415.1567
31	29.95	1-linoleylglycero-2-phospho-ethanolanine	C_23_H_44_O_7_NP	476.5684	476.5653	M – H	0.0031	140.0180, 196.0372
								214.8795, 279.2330
32	38.06	Lysophosphatidic acid	C_21_H_41_O_7_P	435.2514	435.2506	M – H	0.0008	78.9573, 152.9945
33	42.19	Soya-cerebroside I	C_40_H_75_NO_9_	712.5367	712.5358	M – H	0.0009	179.0551, 271.2278
								278.2481, 296.2603
								532.4733, 550.4841
34	42.9	Linoleic acid phosphatidic acid	C_21_H_39_O_7_P	433.4959	433.4974	M – H	-0.0015	78.9574, 152.9946
35	45.84	4-(2-dodecyl)-benzene-sulfonate	C_18_H_30_O_3_S	325.4846	325.4884	M – H	-0.0038	119.0488, 170.0033
								183.0112
36	51.25	4-(1-methyl-dodecyl)-benzene-sulfonic acid	C_19_H_32_O_3_S	339.5191	339.5150	M – H	0.0041	79.9554, 119.0487
								170.0030, 225.0585
								239.0742

**Table 2:** Identificati1on analysis of chemical constituents of *T. hemsleyanums* in positive ion mode of mass spectrometry.

No.	tR (min)	Name	Formula	Detected m/z	Expected m/z	M + X	Error (ppm)	Fragmentor information
37	1.34	Catechins	C_15_H_14_O_6_	291.0851	291.0863	M + H	0.0012	123.0438, 161.0591
								179.0696, 249.0744
								273.0750
38	1.69	Epicatechin	C_15_H_14_O_6_	291.0845	291.0863	M + H	-0.0018	139.0384, 207.0644
								231.0638, 249.0741
								273.0740
39	1.9	Palmitic acid	C_16_H_32_O_2_	257.2466	257.2475	M + H	-0.0009	137.0592, 151.0750
								165.0904, 221.1161
40	3.45	Vitexin-rhamnoside	C_27_H_30_O_14_	579.1687	579.1708	M + H	-0.0021	313.0692, 415.1007
								433.1111, 514.5942
41	4.38	Isoquercetin	C_21_H_20_O_12_	465.1020	465.1028	M + H	-0.0008	285.0382, 303.0485
								363.7647, 395.0804
42	6.32	Orientin	C_21_H_20_O_11_	449.1051	449.1078	M + H	-0.0027	329.3295, 359.1488
43	6.42	Kaempferol-3-O-rutone	C_27_H_30_O_15_	595.1646	595.1658	M + H	-0.0012	287.0537, 352.4147
								433.1127, 449.1062
								545.0139
44	7.61	Isoorientin	C_21_H_20_O_11_	449.1048	449.1078	M + H	-0.0030	287.0537, 337.0678
								395.1428, 431.1667
45	23.06	Gallocatechin	C_15_H_14_O_7_	307.1888	307.0812	M + H	0.1076	163.0748, 261.1832
								271.1683, 285.0766
46	24	Epigallocatechin	C_15_H_14_O_7_	307.1886	307.0812	M + H	0.1074	135.0799, 173.0954
								243.1731, 261.1839
								271.1682, 289.1786
47	24.58	Vitexin	C_21_H_20_O_10_	433.2325	433.1129	M + H	0.1196	293.1132, 317.2460
								335.2565, 399.2344
48	25.45	Taraxerone	C_30_H_48_O	425.3756	425.3778	M + H	-0.0022	217.1945, 283.1677
								339.2303, 365.1367
								406.31027
49	27.26	Isovitexin	C_21_H_20_O_10_	433.1131	433.1129	M + H	0.0002	279.23077, 301.90817
								317.24625, 335.25653
								415.2227
50	29.46	Linoleic acid	C_18_H_32_O_2_	281.2442	281.2475	M + H	-0.0033	245.2253, 263.2358
51	31.19	Methyl linoleate	C_19_H_32_O_2_	293.2097	293.2475	M + H	-0.0378	197.1319, 215.1786
								229.1945, 247.2046
								257.1888, 275.1994

**Table 3:** Linear eq1uation, linear range, correlation coefficient, and detection limit of 10 constituents.

Compound	Regression equation	Linear range (*μ*g/ml)	Correlation coefficient (*r*^*2*^)	Detection limit (pg)	Quantification limit (pg)
Rutin	y = 0.025x -1.099	7.125-1320	0.9924	0.1113	14.4926
Kaempferol	y = 0.062x + 1.015	1.828-58.50	0.9961	0.9141	0.9290
Astragalin	y = 0.111x – 2.688	6.888-1100	0.9971	0.1074	7.4910
Quercitrin	y = 6.393x – 196.137	8.313-1330	0.9992	2.0781	9.5661
Quercetin	y = 7.378x – 292.807	3.344-1070	0.9992	0.1045	9.2285
Vitexin-rhamnoside	y = 0.000436x + 0.0240	1.735-58	0.9997	1.7344	15.5142
Isorhamnetin	y = 0.00673x + 0.152	1.688-54	0.9954	0.4531	11.6001
Vitexin	y = 3.629x + 0.000187	4.125-132	0.9978	2.0625	3.5940
Emodin-8-O-*β*-D-glucopyranoside	y = 0.000982x + 0.0139	1.813-116	0.9986	0.4531	2.7285
Isoquercetin	y = 0.00313x + 0.0602	3.625-116	0.9955	0.1133	6.1772

**Table 4:** Summary of the precision and RSD results of UPLC-Q-Exactive/MS method for the analyzed quality control samples.

Compounds	Intraday precision		Interday precision		Stability		Repeatability	
	accuracy (%)	RSD (%)	accuracy (%)	RSD (%)	accuracy (%)	RSD (%)	accuracy (%)	RSD (%)
Rutin	98.69-103.22	1.93	98.17-102.44	1.65	98.35-102.05	1.41	98.61-101.84	1.16
Kaempferol	98.03-102.13	1.851	98.82-104.04	1.15	95.29-103.09	2.32	96.06-103.27	2.67
Astragalin	98.07-102.47	2.16	96.75-101.24	2.38	94.12-103.32	3.42	97.48-102.65	2.22
Quercitrin	98.57-102.75	2.02	97.83-100.86	1.33	98.72-102.71	1.52	98.15-102.63	2.02
Quercetin	99.83-102.21	0.35	95.02-102.90	1.93	98.37-101.95	1.45	97.48-103.39	1.94
Vitexin-rhamnoside	97.68-101.00	1.30	98.66-101.14	0.72	97.68-100.99	1.30	98.19-100.88	1.08
Isorhamnetin	99.96-100.02	0.03	97.95-103.99	1.90	99.23-100.46	0.53	99.27-100.40	0.50
Vitexin	99.80-100.48	0.25	95.65-103.60	2.81	99.80-100.54	0.28	99.83-100.50	0.26
Emodin-8-O-*β*-D-glucopyranoside	95.62-103.90	2.79	98.83-103.82	2.53	95.05-102.81	2.75	96.30-103.59	2.69
Isoquercetin	97.60-103.10	2.30	96.68-101.86	2.13	97.41-102.98	2.54	97.19-102.74	2.39

**Table 5:** The rate of recovery for 10 constituents (n = 6).

Compounds	Addition level	Average recovery rate (%)	RSD (%)
Rutin	1:0.8	96.95	0.02
	1:1	98.61	2.83
	1:1.2	96.25	1.12
Kaempferol	1:0.8	98.20	2.88
	1:1	97.67	1.21
	1:1.2	98.71	2.05
Astragalin	1:0.8	98.15	0.14
	1:1	99.62	1.10
	1:1.2	98.56	0.67
Quercitrin	1:0.8	99.90	0.41
	1:1	98.58	0.92
	1:1.2	96.59	1.76
Quercetin	1:0.8	97.68	0.18
	1:1	96.06	0.82
	1:1.2	97.75	1.30
Vitexin-rhamnoside	1:0.8	96.62	2.09
	1:1	95.67	2.41
	1:1.2	96.03	1.32
Isorhamnetin	1:0.8	97.13	0.29
	1:1	96.00	1.18
	1:1.2	97.20	0.24
Vitexin	1:0.8	96.24	1.44
	1:1	99.18	1.80
	1:1.2	99.27	1.59
Emodin-8-O-*β*-D-glucopyranoside	1:0.8	97.10	0.33
	1:1	94.86	0.96
	1:1.2	97.99	0.39
Isoquercetin	1:0.8	95.38	0.56
	1:1	98.56	0.67
	1:1.2	97.77	0.62

**Table 6:** The content of 10 constituents in *T. hemsleyanums* ($\overset{-}{x}$  ±  S, average ± standard deviation) (mg/g, n = 3).

Batch	Rutin	Kaempferol	Astragalin	Quercitrin	Quercetin	Vitexin-rhamnoside	Emodin-8-O-*β*- D-glucopyranoside	Isoquercetin	Isorhamnetin	Vitexin
ZJ1	0.0858±0.0011	0.0100±0.0190	0.0446±0.0000	0.0899±0.0006	0.0565±0.0010	-	0.0087±0.0010	0.0345±0.0034	0.0276±0.0060	0.1096±0.0047
FJ3	0.0887±0.0002	0.0102±0.0113	0.0453±0.0002	0.0903±0.0013	0.0584±0.0003	-	0.0132±0.0052	0.0359±0.0074	0.0330±0.0019	0.1107±0.0002
YN2	0.0901±0.0011	0.0393±0.0206	0.0434±0.0003	0.0880±0.0003	0.0615±0.0022	-	0.0154±0.0119	0.0380±0.0099	0.0311±0.0008	0.0324±0.0013
ZJ2	0.1036±0.0022	0.0388±0.0050	0.0445±0.0002	0.0998±0.0002	0.0628±0.0003	-	0.0179±0.0178	0.0383±0.0054	0.0342±0.0011	0.0519±0.0016
ZJ3	0.0905±0.0061	0.0865±0.0436	0.0438±0.0002	0.0907±0.0016	0.0630±0.0001	0.0010±0.0015	0.0182±0.0116	0.0522±0.0061	0.0236±0.0027	0.0262±0.0012
GX3	0.1012±0.0041	0.0498±0.0085	0.0640±0.0004	0.0953±0.0013	0.0809±0.0006	0.0016±0.0002	0.0192±0.0047	0.0576±0.0041	0.0373±0.0071	0.7581±0.0324
FJ2	0.1166±0.0028	0.0979±0.0066	0.0479±0.0274	0.0957±0.0004	0.0756±0.0013	-	0.0210±0.0075	0.0616±0.0028	0.0344±0.0006	0.1145±0.0010
YN3	0.0967±0.0037	0.0867±0.0128	0.0493±0.0001	0.1351±0.0024	0.0577±0.0002	-	0.0209±0.0249	0.0588±0.0037	0.0319±0.0006	0.2680±0.0601
HB3	0.1142±0.0016	0.1016±0.1173	0.0532±0.0002	0.1092±0.0005	0.0750±0.0039	-	0.0224±0.0133	0.1324±0.0016	0.0315±0.0007	-
GZ2	0.1157±0.0031	0.1104±0.0023	0.0583±0.0494	0.1196±0.0006	0.0803±0.0011	0.0008±0.0011	0.0282±0.0058	0.0802±0.0031	0.0597±0.0017	0.2951±0.0072
GX1	0.0970±0.0044	0.1146±0.0268	0.0527±0.0002	0.1014±0.0004	0.0716±0.0003	-	0.0268±0.0022	0.1488±0.0044	0.0331±0.0071	0.0388±0.0015
GZ3	0.0989±0.0201	0.1243±0.0149	0.0572±0.0002	0.1095±0.0000	0.0777±0.0048	0.0027±0.0024	0.0291±0.0133	0.0726±0.0095	0.0318±0.0010	0.0426±0.0007
GX2	0.1121±0.0014	0.0867±0.0004	0.0552±0.0001	0.1058±0.000	0.0627±0.0002	0.0152±0.0064	0.0298±0.0073	0.0711±0.0049	0.0353±0.0011	0.0371±0.0052
FJ1	0.1185±0.0004	0.1379±0.0006	0.0599±0.0004	0.1238±0.0004	0.0759±0.0015	0.0216±0.0041	0.0305±0.0030	0.1068±0.0025	0.0349±0.0041	0.0479±0.0055
HB1	0.1152±0.0187	0.1285±0.0133	0.0572±0.0002	0.1279±0.0009	0.0754±0.0004	0.0305±0.0113	0.0255±0.0020	0.0792±0.0212	0.0338±0.0164	0.2927±0.0045
HB2	0.1296±0.0002	0.2757±0.0179	0.0576±0.0002	0.1435±0.0003	0.1463±0.0002	0.0280±0.0100	0.0300±0.0045	0.1927±0.0425	0.0347±0.0021	0.0005±0.0020
GZ1	0.1374±0.0002	0.2910±0.0210	0.0702±0.0003	0.1443±0.0003	0.0920±0.0002	0.0325±0.0394	0.0335±0.0009	0.1940±0.0981	0.0505±0.0006	0.6811±0.0307
YN1	0.1489±0.0003	0.2967±0.1511	0.0761±0.0003	0.1567±0.0000	0.0960±0.0003	0.0338±0.0045	0.0439±0.0199	0.2207±0.1549	0.0543±0.0037	0.7079±0.0286

- means below the detection limit.

**Table 7:** Summary of results about the components of *T. hemsleyanums* and antiviral inhibition.

Variable	Compound	Correlation Coefficient	P
*X1*	Rutin^*∗*^	0.5470	0.0190
*X2*	Kaempferol*∗*	0.5800	0.0120
*X3*	Astragalin*∗*	0.7110	0.0010
*X4*	Quercitrin*∗*	0.6170	0.0060
*X5*	Quercetin*∗*	0.6140	0.0070
*X6*	Kaempferol-3-O-rutinoside	0.514	0.029
*X7*	Procyanidin dimmer	0.503	0.033
*X8*	Vitexin*∗*	-0.5370	0.0220
*X9*	Epicatechin	0.6410	0.0040
*X10*	Isorhamnetin-3-pyranose arabinose-7-glucosyl rhamnoside	-0.6630	0.0030

*∗*Quantitative compounds.

## Data Availability

Most of the data (Figures 1to 5 and Tables 1to 7) used to support the findings of this study are included within the article. Some of the data used to support the findings of this study are included within the supplementary information file.

## Supplementary Materials

Supplementary Materials Table 1: the information of* T*.* hemsleyanums*. Table 2: parameters of LC-Q-Exactive /MS analysis for 10 constituents. Figure 1: total ion chromatogram of 18 batches of* T*.* hemsleyanum*.
