# Supplementary material for: Qualitative and Quantitative Analysis for the Chemical Constituents of Tetrastigma hemsleyanum Diels et Gilg Using Ultra-High Performance Liquid Chromatography/Hybrid Quadrupole-Orbitrap Mass Spectrometry and Preliminary Screening for Anti-Influenza Virus Components
Source: Evid Based Complement Alternat Med. 2019 Feb 18;2019:9414926. doi: 10.1155/2019/9414926 (PMC6398048; doi:10.1155/2019/9414926)
Supplement: Supplementary Materials — Table 1: the information of T. hemsleyanums. Table 2: parameters of LC-Q-Exactive /MS analysis for 10 constituents. Figure 1: total ion chromatogram of 18 batches of T. hemsleyanum. [file 9414926.f1.pdf]

Table 1 The information of *T. hemsleyanums*

Number	Origin	Batch
ZJ1	Zhejiang	20170923
ZJ2	Zhejiang	20171016
ZJ3	Zhejiang	20170317
GX1	Guangxi	20160924
GX2	Guangxi	20170923
GX3	Guangxi	20170320
YN1	Yunnan	20170927
YN2	Yunnan	20140517
YN3	Yunnan	20160926
HB1	Hubei	20171012
HB2	Hubei	20160925
HB3	Hubei	20171009
FJ1	Fujian	20171015
FJ2	Fujian	20171023
FJ3	Fujian	20170101

GZ1	Guizhou	20160993
GZ2	Guizhou	20161034
GZ3	Guizhou	20170921

TABLE 2 Parameters of LC−Q-Exactive /MS analysis for 10 constituents

Compounds	Precursor ion (m/z)	Product ion (m/z)	Retention time(min)
Rutin	609.5*	301.0; 283.0	4.18
Kaempferol	285.2*	239.0; 211.0	21.4
Astragalin	447.3	285.0*; 227.0	7.9
Quercitrin	447.3	301.0*; 151.0	6.53
Quercetin	301.0*	245.2; 178.9	19.27
Vitexin Rhamnoside	577.5*	457.1; 311.0	4
Isorhamnetin	315.2*	283.0; 255.0	21.64
Vitexin	431.0	341.1*; 311.1	19.77
Emodin-8-o-β-D-glucoside	431.0	353.0*; 311.0	20.62
Isoquercetin	463.3*	301.0; 271.0	4.86

*Quantitative ion

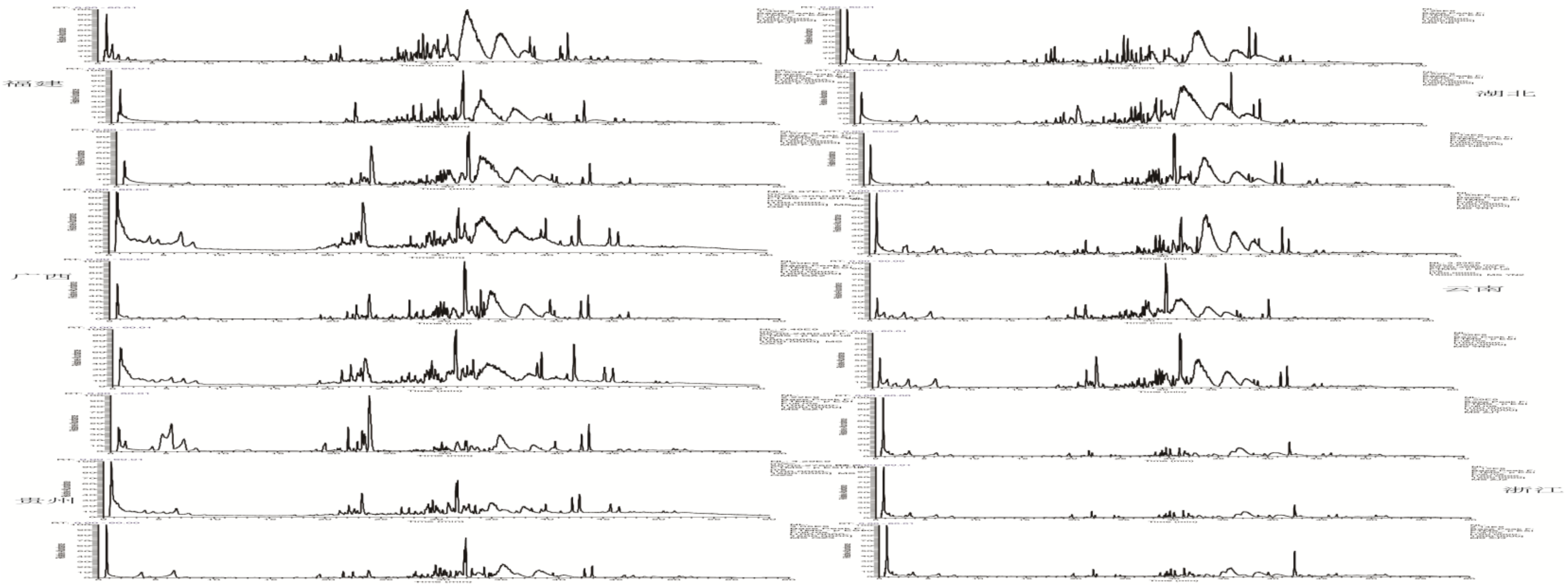

Fig. 1 Total ion chromatogram of 18 batches of T. *hemsleyanum*
